# Sesquiterpenes and monoterpenes from different varieties of guava leaf essential oils and their antioxidant potential

**DOI:** 10.1016/j.heliyon.2022.e12104

**Published:** 2022-12-07

**Authors:** Shanthirasekaram Kokilananthan, Vajira P. Bulugahapitiya, Harshi Manawadu, Chinthaka Sanath Gangabadage

**Affiliations:** Department of Chemistry, Faculty of Science, University of Ruhuna, Matara 81000, Sri Lanka

**Keywords:** Antioxidants, Chemical compositions, Essential oil, Guava varieties, Gas chromatography-mass spectrometriy

## Abstract

Despite that Sri Lanka is a biodiversity hotspot with numerous guava varieties (*Psidium guajava* L.), no adequate scientific research has been reported on leaf essential oil (EO) composition based on varieties and its pharmacological properties, namely antioxidant properties. Therefore, this study focused to evaluate the chemical compositions and antioxidative capacity of EOs isolated from leaves of seven guava varieties grown in Sri Lanka, including apple-guava (*P. pomiferum,* PGA), common-guava (*P. guajava,* PGCG), two wild-guava; cultivar of *P. guajava* (PGG) and a cultivar of *P. guineense* (PGE), two introduced varieties of *P. guajava* (PGK and PGP), and one introduced variety of *P. guineense* (PGC). The EOs were isolated using hydro-distillation and the chemical compositions were analyzed using Gas Chromatography-Mass Spectrometry (GC-MS) technique, and the compounds that showed greater than 90% matching value were considered for characterization. The yields of EOs ranged from 0.02-0.26% (w/w) where PGE produced the greatest amount. About sixty-eight chemical compounds were identified from seven varieties and Sesquiterpenes were found to be the most abundant in the PGCG, PGG, PGE, and PGA varieties, whereas monoterpenes were found to be the most abundant in PGK, PGP, and PGC varieties. The sesquiterpenes, Nerolidol (70.0–7.9%), (-)-Globulol (21.0–7.0%), and Caryophyllene (20.4–1.4%) and monoterpenes, D-Limonene (30.3–14.1 %) were found as the major compounds of all studied guava varieties. Twenty-eight compounds were identified for the first time in guava EOs, including Cadinadiene-1,4, Benzylacetaldehyde, and Epiglobulol. The antioxidant efficacy of EOs varied from 329.56 ± 2.01 to 85.70 ± 2.01 μL Trolox Eq/L, where PGE showed the highest antioxidative potential. Ultimately, the chemical constituents and antioxidant capacity of isolated EOs varied with the variety, with EO from PGE leaves exerting an amazing antioxidant capacity compared to the others and being rich in Nerolidol. The findings of this study fill the gap in the literature on chemical constituents in the EO of guava leaves, and also it will open the avenue to discover novel potential compounds with outstanding pharmacological activities from guava leaves.

## Teaser

Antioxidants; Chemical compositions; Essential oil; Guava varieties; Gas chromatography-mass spectroscopy analysis.

## 1 Introduction

The medicinal tree, *Psidium guajava* L. (Myrtaceae), is endemic to South America and has been mainly distributed in tropical and subtropical regions, such as East Asia, Central and South America, and South Africa [1]. It is commonly known as guava, and it has long been utilized in the treatment of a variety of illnesses [2]. Since guava leaves are abundant in essential phytochemicals, they have remarkable pharmacological properties such as antioxidant, antitussive, antiallergic, antidiarrheal, anti-inflammatory, antinociceptive, antimicrobial, hepatoprotective, antidiabetic, and so on [3].

The biological effects of *P. guajava* leaves are often related to their essential oils (EOs) which are the major components of the leaves. Many compounds, particularly terpenoids, including caryophyllene, α-pinene, limonene, nerolidol, farnesene, veridiflorol, calamenene, α-cadinene, caryophyllene, α-cubebene, and aromadendrene, are often identified from the EOs isolated from guava leaves all over the world [1, 4, 5, 6, 7, 8]. Exogenous factors such as precipitation, light, season, altitude, and soil properties are shown to be influenced the composition of EOs. Furthermore, endogenous variables such as anatomical, physiological, and genetic factors can alter the qualitative or quantitative levels of the EOs' chemical components [9]. Despite the fact that numerous studies have been conducted on the chemical compositions of EO isolated from the leaves of common guava varity in the many part of the world [1, 4, 5, 6, 7, 8], few studies have been conducted on comparison with EOs from other guava varieties [9].

Sri Lanka has many guava varieties, and these can be classified as commonly cultivated, wild, and introduced varieties. In particular, *P. guajava* (Common-guava, PGCG), *P. cattleyanum* (Strawberry-guava), *P. pomiferum* (Apple-guava, PGA), two types of wild guava (Getta-pera, PGG – a cultivar of *P. guajava*; Embul-pera, PGE – a cultivar of *P. guineense*), and many introduced varieties (Kanthi, *P. guajava,* PGK; Pubudu, *P. guajava*, PGP; and Costorican, a cultivar of *P. guineense,* PGC) and some more varities are available in Sri Lanka [10, 11]. Even though guava leaf EO is an important source for many biologically active terpenoids, no research has been focused on the variation of chemical composition in leaf EO based on the varieties. Therefore, this research was intended to fill that gap by investigating seven guava varieties grown in Sri Lanka with the goal of developing chemical profiles of EOs in order to identify novel compounds and determine the antioxidative potential of EOs for the use in future studies on pharmacological activities and healthcare applications.

## 2 Materials and methodology

### 2.1 Materials

#### 2.1.1 Plants

**Figure 1:**
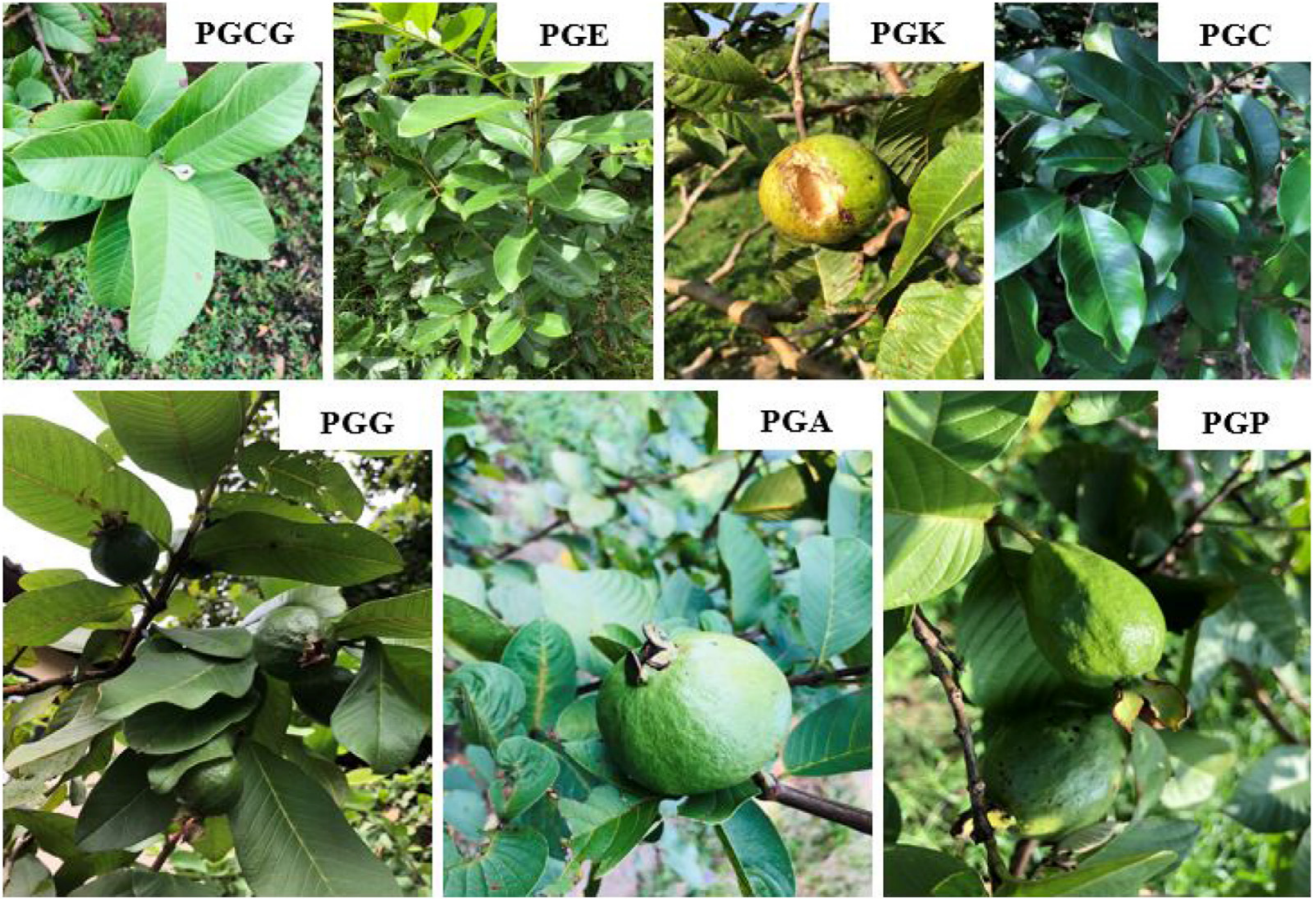
The pictures of the seven selected guava varieties for this study. (PGCG: Common-guava, PGG: Getta-pera, PGE: Embul-pera, PGA: Apple-guava, PGK: Kanthi, PGP: Pubudu, PGC: Costorican).

Fresh leaves of all seven selected guavas (each 1 kg) were collected from various locations in Sri Lanka. Table 1 and Figure 1 disclose the details and images for all the selected guava varieties.

**Table 1:** Detailed information on collected guava leaves for this study.

Common Name	Scientific Name	Abbreviation	Location	Latitude (ºN)	Longitude (ºE)
Common guava	*P. guajava*	PGCG	Home garden in Matara, Sri Lanka	5.9478	80.5483
Getta-pera	*P. guajava*	PGG			
Embul-pera	*P. guineense*	PGE			
Apple-guava	*P. pomiferum*	PGA	Lake Serenity Resort & Spa, Gonapitiya Road, Ratnapura, Sri Lanka	6.7397	80.4698
Kanthi	*P. guajava*	PGK	Fruit Crops Research and Development Centre, Department of Agriculture, Horana, Sri Lanka	6.7166	80.0620
Pubudu	*P. guajava*	PGP			
Costorican	*P. guineense*	PGC

#### 2.1.2 Chemicals

Acetic acid (CH_3_COOH, SIGMA-ALDRICH, AR grade, 99.8%), Diethyl ether (SIGMA-ALDRICH, GC grade, 99.8%), Ferric chloride hexahydrate (FeCl_3_·6H_2_O, CDH, AR grade, 98.0%), Hexane (SIGMA-ALDRICH, GC grade, 99%), Hydrochloric acid (HCl, CDH, AR grade, 35.40%), (±)-6-Hydroxy-2,5,7,8-tetramethylchromane-2-carboxylic acid (Trolox, SIG-MA-ALDRICH, AR grade, 97%), Sodium acetate trihydrate (CDH, AR grade, 99.5 %)**,** Sodium sulphate anhydrous (Na_2_SO_4_, CDH, AR grade, 99.5%) and 2,4,6-Tripyridyl-S-triazine (TPTZ, SIGMA-ALDRICH, AR grade, 99%) were used.

### 2.2 Methodology

#### 2.2.1 Sample preparation

All collected leaves were maintained in the dark at 4 °C immediately after collection before being brought to the laboratory. The guava leaves were washed thoroughly with running water, then with distilled water and the washed leaves were crushed using a grinder (Philips mixer grinder HL 7756 09).

#### 2.2.2 Extraction of essential oils

**Figure 2:**
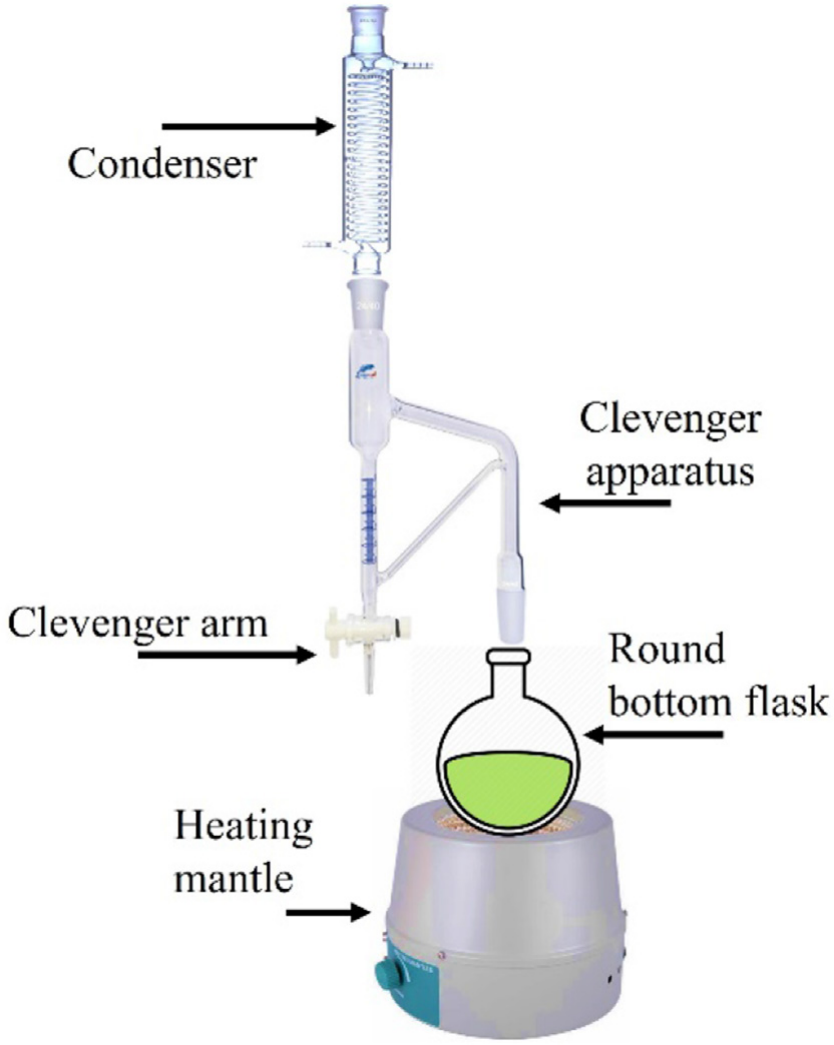
Hydro-distillation setup (Clevenger apparatus).

The crushed leaves (200.00 g) were introduced into a round-bottom flask into which distilled water (1000.0 ml) was added (plant material to distilled water ratio = 1:5 w/v) [12]. The flask was connected to the Clevenger type apparatus that was connected to the condenser and placed on the heating mantle for around 3 h to boil at 100 °C. The distillation rate was maintained at around twenty-five drops per minute, and the continually condensed distillate was collected to a vial in the receiver arm of the Clevenger apparatus after heating for around 3 h to guarantee the optimal yield (Figure 2 illustrate the hydro-distillation apparatus). Obtained EOs were dehydrated by adding anhydrous Na_2_SO_4_, and the water-free oil was stored in a sealed amber color vial in the refrigerator at 4 °C for testing with sensitive GC-MS. The hydro-distillation procedure was done trice (3 × 200.00 g) to ensure reproducibility of EO yields. By dividing the weight of water-free EO by the weight of ground plant materials before distillation, the percentage yield of EO was calculated [5].

#### 2.2.3 Gas chromatography – mass spectrometry analysis of essential oils

Each EO sample (0.5 mL) was dissolved in 10.0 mL of GC grade *n*-Hexane. PTFE membrane filter paper (pore size: 0.45 μm) was used to filter the dehydrated samples. The filtered samples (1.0 μL) were injected into the GC-MS. The GC–MS analysis was performed on an Agilent 7890A series gas chromatograph interfaced to an Agilent 5975C series MSD version mass selective detector (Model No: 5975C TAD inert XL EI/CI MSD, Agilent Technologies, Palo Alto, CA, USA). Agilent 19091S–433HP-5MS 5% Phenyl Methyl Silox, 325 °C: 30 m × 250 μm x 0.25 μm column was used. The chromatography conditions were as follows (gradient): initial oven temperature: 70 °C for 4 min then increasing by 8 °C/min to 270 °C and then kept stable for 10 min, run time-39 min; carrier gas: helium at a flow rate of 1 ml/min; injection volume: 1.0 μL in split mode. The conditions of the mass unit: ion source temperature 230 °C; mass spectrum recorded at an ionization voltage of 1624 with a mass scan range of 33–550 m/z.

#### 2.2.4 Identification of constituents in essential oils

Individual compounds of all selected guava EOs were identified using mass spectra obtained in GC-MS and by comparing the obtained mass spectral data with the NIST 08 and NIST Chemistry WebBook (NIST Standard Reference Database Number 69), and by referring to the literature [13]. The chemical constituents with a matching value of greater than 90% were considered in this study.

#### 2.2.5 Antioxidant analysis (ferric reducing antioxidant power (FRAP) assay)

The FRAP assay of EO was carried out using standard methods reported in the literature [14, 15, 16]. About 3.0 mL of freshly made FRAP reagent [300 mM acetate buffer (pH-3.6): 10 mM TPTZ (in 40 mM HCl): 20 mM FeCl_3_ in a 10:1:1 ratio] was mixed with 100 μL of sample solution. The absorbance at 593 nm was measured after 30 min of incubation at 37 °C using the spectrophotometer (HITACHI, UH5300). Trolox was used as the standard.

#### 2.2.6 Statistical analysis

Analysis of variance (ANOVA) and T-test (LSD) (LSD-Least Significant Difference) was used to analyze and compare the data. SAS OnDemand for Academics: Studio (SAS 9.4) software was used for the statistical analysis. The data were presented in the form of means and standard deviations.

## 3 Results and discussion

### 3.1 Isolation of essential oils from selected seven guava varieties

**Figure 3:**
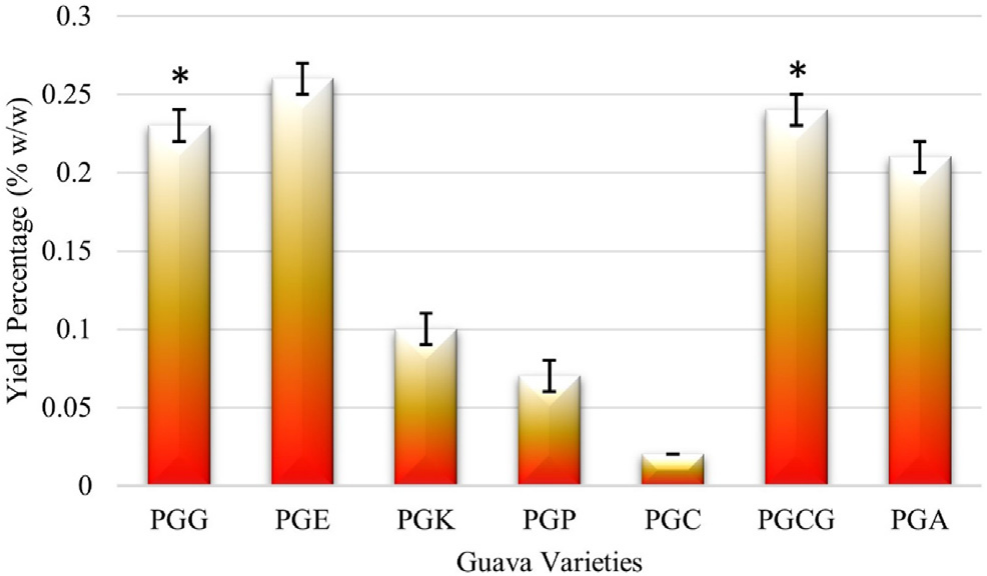
Comparison of yields of isolated EOs by hydro-distillation of seven guava varieties (∗: no differences at 5% significant level).

**Figure 4:**
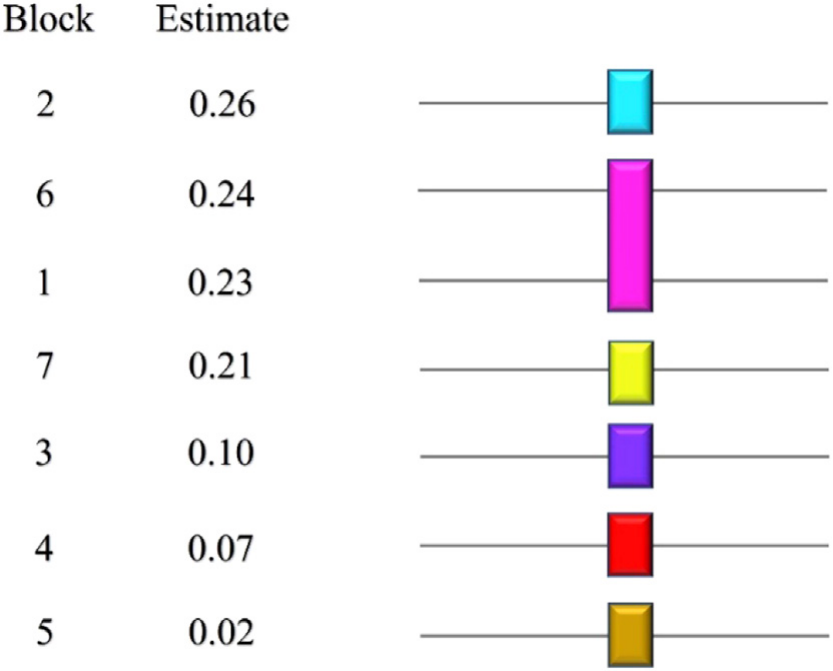
T-test (LSD) for yields of isolated EOs of seven guava varieties. (α = 0.05, guava varieties: 1: PGG, 2: PGE, 3: PGK, 4: PGP 5: PGC, 6: PGCG, and 7: PGA, Estimate: mean value of essential oil extraction yields, means covered by the same bar are not significantly different).

The isolated EOs of seven guava varieties were shown to be yellowish in color, but the intensity of the color varied with the variety, and had a distinct and powerful odor (the aroma of PGE leaves' EO was stronger than that of other kinds). The hydro-distillation technique yielded EOs in the range of 0.02 ± 0.00 to 0.26 ± 0.01% w/w on a fresh leaves weight basis, as shown in Figure 3. When compared to all other guava varieties, the EO of PGE leaves was higher. As shown in Figure 4, the statistical analysis revealed that the same, and significantly different at the 5% significant level. Furthermore, at the 5% significant level, the yield of PGG and PGCG EOs was the same, as shown in Figure 4. In comparison to some previous findings [5, 8, 17, 18], the yields are comparable, and the minor variance may be due to the seasonal change or growing habitats, and the most significantly the quantity and the chemical compositions may vary with the varieties.

### 3.2 Chemical profiles of the EOs from seven guava varieties

**Figure 5:**
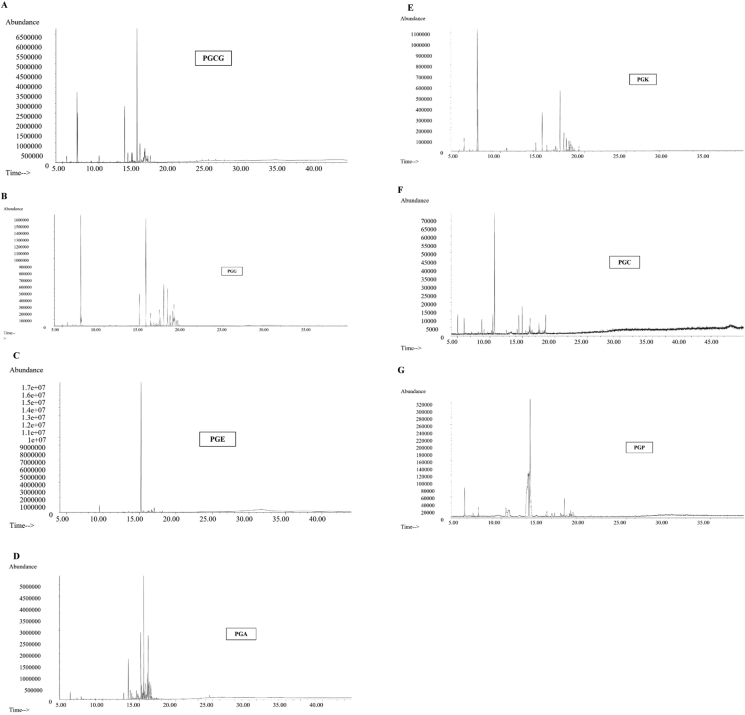
The GC spectra of all seven essential oils extracted from guava varieties. (A) GC spectra of PGCG leaves' EO, (B) GC spectra of PGG leaves' EO, (C) GC spectra of PGE leaves' EO, (D) GC spectra of PGA leaves' EO, (E) GC spectra of PGK leaves' EO, (F) GC spectra of PGC leaves' EO, (G) GC spectra of PGP leaves' EO.

Despite the fact that GC-MS analysis showed a large number of chemical constituents in each EO of guava leaves, this finding only considered matches with the NIST MS library that were more than 90%. Based on the matching value, 68 chemical compositions were identified which are tabulated in detail as shown in Table 2. Figure 5 depicts the GC spectra of all seven EOs extracted from guava varieties.

**Table 2:** Chemical compositions of isolated essential oils of seven guava varieties leaves by Gas Chromatography-Mass Spectrometry (GC-MS) analysis.

No	Chemical Name	RT (min)	MF	MW (g/mol)	Relative percentage (%)						
					A	B	C	D	E	F	G
**1**	α-Pinene^g^	5.01	C_10_H_16_	136.23	0.2			0.3			8.0
**2**	Benzaldehyde^e,f^	5.56	C_7_H_6_O	106.12	1.5		1.5	0.9	3.6	3.6	
**3**	β-Myrcene	6.16	C_10_H_16_	136.23	0.3			0.4			
**4**	cis-3-Hexenyl acetate	6.66	C_8_H_14_O_2_	142.20		0.1					
**5**	Isodurene∗	6.90	C_10_H_14_	134.22	0.1						
**6**	D-Limonene^a,d,e^	7.00	C_10_H_16_	136.23	14.1			21.4	30.3		
**7**	Eucalyptol^a,e^	7.05	C_10_H_18_O	154.25	9.7	0.3	0.5		10.6	1.0	
**8**	1,3,8-p-Menthatriene∗	7.12	C_10_H_14_	134.22				0.1			
**9**	β-(E)-Ocimene	7.13	C_10_H_16_	136.23	0.1		0.2				
**10**	Crithmene∗	7.58	C_10_H_16_	136.23	0.2						
**11**	ß-Ocimene	7.63	C_10_H_16_	136.23		0.1	0.1				
**12**	Limona ketone∗	8.95	C_9_H_14_O	138.21	0.2		0.2				
**13**	L-4-Terpineol	9.79	C_10_H_18_O	154.25	0.2						
**14**	Benzylacetaldehyde∗^b^	10.15	C_9_H_10_O	134.17		3.7	0.1				
**15**	Terpilene∗	12.42	C_10_H_16_	136.23	0.1						
**16**	Neryl acetate	12.57	C_12_H_20_O_2_	196.29	0.3						
**17**	1-Phenylcyclopropane∗	12.70	C_9_H_10_	118.18		0.2					
**18**	Caryophyllene^a,c,d,e^	13.49	C_15_H_24_	204.35	10.0	1.4	6.1	20.4	9.5		
**19**	β-Farnesene	13.83	C_15_H_24_	204.35	0.2						
**20**	(E)-β-Farnesene	13.83	C_15_H_24_	204.35		0.2					
**21**	1,4,7,-Cycloundecatriene, 1,5,9,9-tetramethyl-, Z,Z,Z-	13.94	C_15_H_24_	204.35	1.9			0.6	1.5		
**22**	Allylbenzene∗	14.04	C_9_H_10_	118.18		0.1					
**23**	γ-Muurolene	14.20	C_15_H_24_	204.35	0.4	0.4	0.3	0.7			
**24**	α-Cubebene^d^	14.21	C_15_H_24_	204.35		0.7	1.0	6.1			
**25**	Valencene	14.37	C_15_H_24_	204.35		0.2					
**26**	β-Panasinsene∗	14.37	C_15_H_24_	204.35	0.2		0.1				
**27**	cis-α-Bisabolene	14.46	C_15_H_24_	204.35	1.9						
**28**	γ-Maaliene	14.46	C_15_H_24_	204.35				0.3			
**29**	γ-Selinene	14.47	C_15_H_24_	204.35			0.4				
**30**	β-Bisabolene	14.55	C_15_H_24_	204.35	1.7						
**31**	Butylated hydroxytoluene∗	14.60	C_15_H_24_O	220.35	0.4	0.2	1.3	0.2	0.4		
**32**	D-Germacrene	14.69	C_15_H_24_	204.35			0.2				
**33**	β-Cadinene∗	14.78	C_15_H_24_	204.35	0.4						
**34**	(Z)-Calamenene	14.79	C_15_H_22_	202.33			1.3				
**35**	Nerolidol^a,b,c,d,e^	15.20	C_15_H_26_O	222.37	24.0	70.2	10.0	7.9	13.4		
**36**	Epiglobulol∗^c^	15.29	C_15_H_26_O	222.37			2.2	1.0			
**37**	β-Maaliene∗	15.39	C_15_H_24_	204.35			1.4				
**38**	α-Selinene	15.39	C_15_H_24_	204.35				0.7			
**39**	2,6-Di-tert-butylquinone∗	15.48	C_14_H_20_O_2_	220.31						1.1	
**40**	(-)-Spathulenol	15.51	C_15_H_24_O	220.35			1.6				
**41**	Espatulenol∗	15.51	C_15_H_24_O	220.35				0.7			
**42**	Anozol∗	15.58	C_12_H_14_O_4_	222.24		1.2					
**43**	Caryophyllene oxide^a^	15.59	C_15_H_24_	204.35	3.9						
**44**	(-)-Globulol^c,d^	15.59	C_15_H_26_O	222.37			21.0	7.0			
**45**	Aromadendrene^e^	15.65	C_15_H_24_	204.35			1.7	0.6	3.4		
**46**	Viridiflorol	15.70	C_15_H_26_O	222.37			1.5	0.7			
**47**	β-Selinene^c^	16.06	C_15_H_24_	204.35			4.4	0.5			
**48**	Cadinadiene-1,4∗^,e^	16.08	C_15_H_24_	204.35	1.2	0.8	0.5	1.7	2.8		
**49**	α-Cadinol^e^	16.23	C_15_H_26_O	222.37		0.7			2.3		
**50**	Copaene^a,e^	16.27	C_15_H_24_	204.35	2.9	1.4		1.2	2.0		
**51**	δ-Cadinene	16.58	C_15_H_24_	204.35		1.0					
**52**	(1S)-cis-Calamenene^d^	16.58	C_15_H_22_	202.33				3.6			
**53**	α-Bisabolol^b^	16.67	C_15_H_26_O	222.37		2.2					
**54**	Farnesol^b^	17.01	C_15_H_26_O	222.37	1.5	2.8					
**55**	E,E-Farnesal∗	17.25	C_15_H_24_O	220.35		0.3					
**56**	Ascabiol∗	17.58	C_14_H_12_O_2_	212.24	0.1						
**57**	11-Oxatetracyclo [5.3.2.0(2,7).0(2,8)] dodecan-9-one∗	17.65	C_11_H_14_O_2_	178.23			0.2				
**58**	tau-Muurolol	18.37	C_15_H_26_O	222.37		0.5					
**59**	Methyl palmitate∗	19.08	C_17_H_34_O_2_	270.50		0.2					
**60**	Palmitic acid∗	19.42	C_16_H_32_O_2_	256.42	0.1			0.7			
**61**	Methyl 8-octadecenoate∗	20.77	C_19_H_36_O_2_	296.50		0.2					
**62**	Phytol∗	20.90	C_20_H_40_O_2_	296.50	0.2						
**63**	Adipic acid∗	23.35	C_6_H_10_O_4_	146.14				0.7			
**64**	Tetracosane	24.12	C_24_H_50_	338.70	0.5						
**65**	Hexacosane∗	24.97	C_26_H_54_	366.70	0.4						
**66**	Heptacosane∗	25.96	C_27_H_56_	380.70	0.4						
**67**	Eicosane∗	27.13	C_20_H_42_	282.50	0.5						
**68**	Heneicosane	30.34	C_21_H_44_	296.60	0.2						
**Total**					80.0	89.1	57.8	78.4	79.8	5.7	8.0
**Aldehyde (2,14)**					1.5	3.7	1.6	0.9	3.6	3.6	
**Alkane (64,65,66,67,68)**					2.0						
**Alkene (5)**					0.1						
**Aromatic compounds (17,22)**						0.3					
**Carboxylic acid (63)**								0.7			
**Diterpene alcohol (62)**					0.2						
**Ester (4,16, 42,56,59,61)**					0.4	1.7					
**Ketone (12,39,57)**					0.2		0.4			1.1	
**Lipophilic organic compound (31)**					0.4	0.2	1.3	0.2	0.4		
**Monoterpene (1,3,6,8,9,10,11,15)**					15.0	0.1	0.3	22.2	30.3		8.0
**Monoterpene alcohol (7,13)**					9.9	0.3	0.5		10.6	1.0	
**Fatty acid (60)**					0.1			0.7			
**Sesquiterpene (18,19,20,21,23,24,25,26,27,28,29,30,32, 33,34,37,38,43,45,47,48,50,51,52)**					24.7	6.1	17.4	36.4	19.2		
**Sesquiterpene alcohol (35,36,40,41,44,46,49,53,54,58)**					25.5	76.4	36.3	17.3	15.7		
**Sesquiterpene aldehyde (55)**						0.3

(A: PGCG, B: PGE, C: PGA, D: PGG, E: PGK, F: PGP, G: PGC, RT: Retention time, MF: Molecular formula, MW: Molecular weight, ∗: newly discovered chemical constituents, ^a^: Most prominent chemical constituents found in EO of PGCG, ^b^: Most prominent chemical constituents found in EO of PGE, ^c^: Most prominent chemical constituents found in EO of PGA, ^d^: Most prominent chemical constituents found in EO of PGG, ^e^: Most prominent chemical constituents found in EO of PGK, ^f^: Most prominent chemical constituents found in EO of PGP, ^g^: Most prominent chemical constituents found in EO of PGC).

Based on the GC-MS data of chosen seven guava varieties' EOs, the number of chemical constituents’ availability is as follows; PGCG > PGE > PGG > PGA > PGK > PGP > PGC and the amount of them vary with the varieties. Monoterpenoids and sesquiterpenoids were the most prominent components in the EO of guava leaves. The PGE variety had the largest concentration of sesquiterpenes (82.8%, sesquiterpenes alcohol and aldehyde inclusively), while Nerolidol had the highest proportion (70.2%). The second highest sesquiterpenes content was observed in both PGA and PGG varieties of guava (same quantity in both varieties; 53.7%, sesquiterpenes alcohol inclusively), with (-)-Globulol (21.0%) being abundant in PGA and Caryophyllene (20.4%) being abundant in PGG. The PGK variety, on the other hand, had the largest level of monoterpenes (40.9%, monoterpene alcohols inclusively) with D-Limonene (30.3%) being prevalent.

The PGCG variety showed the highest number of chemical compositions in its EO, including D-Limonene, Eucalyptol, Caryophyllene, and Nerolidol with a high concentration. The PGE was revealed to have the second-highest number of chemical compositions, which is the richest variety with Nerolidol. When it comes to the EO of PGG, which is rich with D-Limonene, Caryophyllene, Nerolidol, and (-)-Globulol. The PGA is shown to have (-)-Globulol, Nerolidol, and Caryophyllene in high concentrations. Monoterpenoids are particularly abundant in introduced varieties such as PGK, PGP, and PGC, while PGK also contains sesquiterpenoids. In particular, PGK contains Nerolidol, Caryophyllene, Eucalyptol, and D-Limonene. PGP has a high concentration of Benzaldehyde, whereas PGC has a high concentration of α-Pinene. Table 2 shows the detailed information on the prominent compositions of the isolated EOs. Many of the varieties used in the study contained Nerolidol, Caryophyllene, and D-Limonene. Most notably, the EO of the seven guava varieties yielded 28 compounds that are previously unknown in guava EOs, those are specifically highlighted in Table 2. The majority of the newly identified chemicals were found in the EO of PGCG leaves in particular.

The literature reports of these firstly identified compounds from guava EOs have shown that they exert important pharmaceutical properties such as, Terpilene has been utilized as an alternate therapy for *Trypanosoma evansi* infection [19] and it has antioxidant activity [20]. Natural butylated hydroxytoluene is a well-known antioxidant [21, 22] whereas cadinadiene-1,4 has been identified as a potential antimalarial agent [23], and ascabiol is used to treat scabies [24]. Phytol is the most important pharmacological molecule, with antioxidant, cytotoxic, antibacterial, anti-inflammatory, immune-modulating, antinociceptive, anxiolytic, apoptosis triggering, metabolism-modulating, and autophagy properties [25]. Some compounds identified in this list have not yet been scientifically investigated based on their pharmacological aspects.

**Figure 6:**
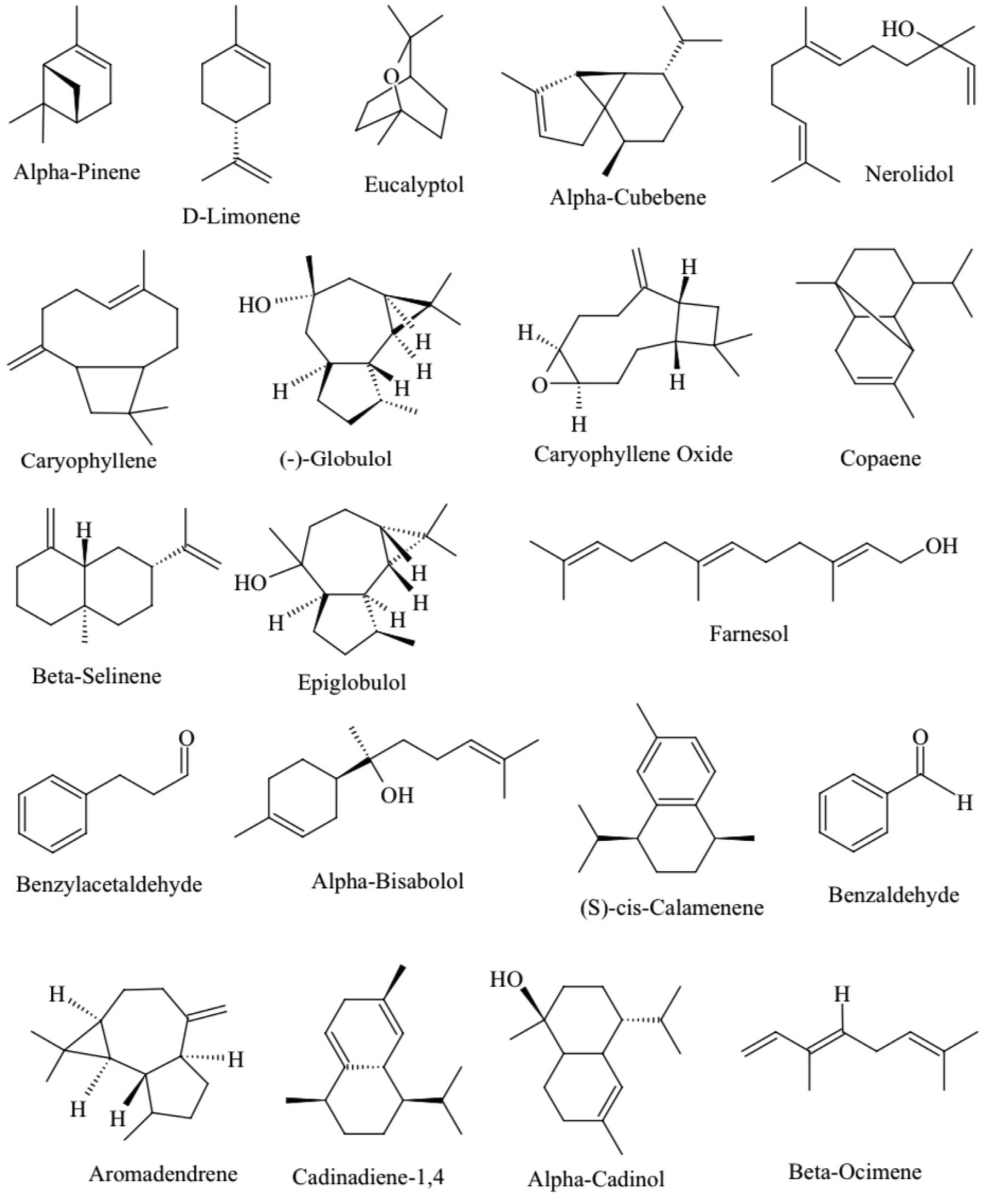
Chemical structures of highly available important chemical constituents in the isolated EOs of seven guava leaves.

Furthermore, when considering the overall chemical constituents, the majority of the compounds identified in each EO have been reported to have specific pharmacological activity. Nerolidol, a chemical found in high concentrations in five guava types out of seven, possesses antioxidant capabilities, antifungal characteristics, antiparasitic activity, and so on [26, 27, 28]. D-Limonene is abundant in the essential oils of PGCG, PGG, and PGK and has antifibrotic, antibacterial, drug-modulatory, and anti-diabetic properties [29, 30, 31]. Taking into account that Caryophyllene, which is found in five of the seven guava varieties and has anti-inflammatory, analgesic, anti-catabolic, and pro-anabolic properties [32, 33]. The compound, Copaene possesses cytotoxic, genotoxic/antigenotoxic, anticarcinogenic, and antioxidant/oxidant properties [34, 35] whereas Cubebene possesses anti-inflammatory, neuroprotective, and antioxidant properties [36, 37, 38] and (-)-Globulol has antibacterial properties [39]. Figure 6 depicts the structures of some key chemical components found in guava EOs. All chosen guava varieties show the presence of a plethora of beneficial chemical components. Each guava variety has distinct characteristics in terms of chemical composition and quantity.

### 3.3 Antioxidant analysis

**Figure 7:**
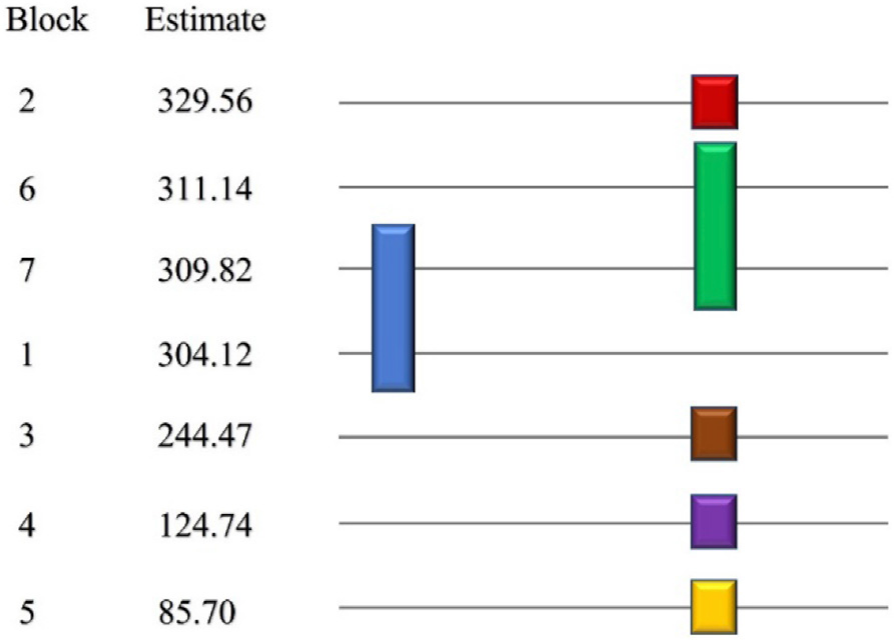
T-test (LSD) for EOs antioxidant capacity of seven guava varieties. (α = 0.05, guava varieties: 1: PGG, 2: PGE, 3: PGK, 4: PGP 5: PGC, 6: PGCG, and 7: PGA, Estimate: mean value of antioxidant capacity, means covered by the same bar are not significantly different).

The FRAP technique is a simple, highly fast, low-cost, and reproducible method for measuring antioxidants in plasma or botanicals. Table 3 depicts the results of ferric reducing capacity determined by the FRAP assay for all EOs isolated from the seven guava varieties. All of the EOs showed ferric reducing ability with varying degrees. In terms of Trolox concentration, the EO of the PGE variety had the highest (p < 0.05) ferric reducing ability (329.56 ± 2.01 μL Trolox Eq/L), followed by the EO of PGCG. The EO of PGC had the lowest ferric reducing capability. Accordingly, Figure 7 depicts EO's antioxidative capacity in the sequence of PGE > PGCG > PGA > PGG > PGK > PGP > PGC. In Figure 7, statistical evidence indicates that the antioxidative capacity of EOs of PGCG, PGA, and PGG is not significantly different at the 5% significant level.

**Table 3:** The total antioxidant capacity of EOs from seven guava varieties based on FRAP assay.

Guava varieties	FRAP value (μL Trolox Eq/L)
PGG	304.12 ± 2.01^b^
PGE	329.56 ± 2.01
PGK	244.47 ± 1.32
PGP	124.74 ± 6.96
PGC	85.70 ± 2.01
PGCG	311.14 ± 1.52^a^
PGA	309.82 ± 3.31^a,b^

(Same English letters in FRAP value express that there is no differences in the total antioxidant capacity at 5% significant level.).

Some other researchers have reported significant antioxidant activity of guava leaf EOs. Lee et al. discovered that the guava leaves EO has a moderate potential source of natural antioxidants, as evidenced by the DPPH assay [40, 41]. The findings from Jassal, et al. and Priyanto, et al. were consistent with what Lee, et al. reported [42, 43]. To emphasize, while there have been some studies available on the EO isolation from guava leaves, specifically commonly grown guava, and its antioxidant capacity, no studies have been conducted with respect to Sri Lankan varieties of guava on comparative basis. According to our understanding, this is the first detailed study based on the antioxidant properties and chemical compositions of EOs in the leaves of Sri Lankan guava varieties. Notably, Joseph and Priya reviewed that the guava leaves EO has other important pharmacological properties such as antimicrobial, antinociceptive, repellent, insecticidal, anticancer, anti-inflammatory effects, and so on [44]. With our noval findings in this work, further studies based on the pharmacological properties of EOs of guava varieties may produce interesting results on varieties basis, as we observed the variation in antioxidant properties on varieties basis. Finally, this research will provide the avanue to isolate novel chemical compounds from guava leaves, further research into the novel pharmacological properties from guava leaves, and the development of a comprehensive library of chemical constituents of guava leaves. Some limitation of this study can be highlighted as the introduced varieties used in the study would be changed by the particular research institute of the country based on their palatability and the adaptability to the different geological conditions in the country.

## 4 Conclusions

This study produced the chemical profiles of EOs isolated from seven guava varieties grown in Sri Lanka. About sixty eights chemical constituents were identified from the EOs of guava varieties, and their availability varies among them. The terpenes, Caryophyllene, and Nerolidol are available in five varieties except in PGP and PGC. Other major compounds found in guava varieties include D-Limonene, Eucalyptol, (-)-Globulol, and α-Pinene. Importantly 28 compounds were identified from the EOs of guava leaves for the first time in this study. Guava EOs exert exceptional antioxidant potential varying with the varieties. All EOs exert higher antioxidant properties. Most of the chemical constituents identified from EOs of guava leaves in this study have been reported to show definite pharmacological activities. Therefore, guava leaves which are discarded as agricultural waste can be utilized for obtaining pharmacologically active compounds as well as developing into noval functional foods and crude drugs.

## Declarations

### Author contribution statement

Shanthirasekaram Kokilananthan: Performed the experiments; Analyzed and interpreted the data; Wrote the paper.

Vajira P. Bulugahapitiya: Conceived and designed the experiments; Contributed reagents, materials, analysis tools or data; Wrote the paper.

Harshi Manawadu, Chinthaka Sanath Gangabadage: Analyzed and interpreted the data; Wrote the paper.

### Funding statement

Professor Vajira P Bulugahapitiya was supported by the World Bank project through the Ministry of Education of Sri Lanka (AHEAD/RA3/DOR/RUH/SCI/CHE-No-05).

### Data availability statement

Data included in article/supplementary material/referenced in the article.

### Declaration of interests statement

The authors declare no conflict of interest.

### Additional information

No additional information is available for this paper.
